# Environmental Impacts of Styrene-Butadiene-Styrene Toughened Wood Fiber/Polylactide Composites: A Cradle-to-Gate Life Cycle Assessment

**DOI:** 10.3390/ijerph16183402

**Published:** 2019-09-13

**Authors:** Tao Qiang, Yaxuan Chou, Honghong Gao

**Affiliations:** 1 School of Materials Science and Chemical Engineering, Xi’an Technological University, Xi’an 710021, China; chouyaxuan@163.com; 2 School of Mechatronic Engineering, Xi’an Technological University, Xi’an 710021, China; gaohonghong@xatu.edu.cn

**Keywords:** wood flour, polylactide, SBS, life cycle assessment, environmental impact

## Abstract

In this study, a life cycle assessment (LCA) was used to investigate the environmental benefits of using styrene-butadiene-styrene (SBS) to modify polylactide (PLA)-based wood plastic composites (WPCs), with a process-based and input–output hybrid model. The results showed that one metric ton of the SBS-modified WPCs required 1.93 × 10^8^ kJ of energy (Sample 2) and 46 m^3^ of water (Sample 4), and that it could produce 42.3 kg of solid waste (Sample 2) during its cradle-to-gate life cycle phases. The environmental impact load (EIL) and photochemistry oxidation potential (PCOP) accounted for the largest share, while the eutrophication potential (EP) took the smallest one. The total EIL index of Samples 1, 2, 3, and 4 added up to 1.942, 1.960, 1.899, and 1.838, respectively. The SBS-modified WPCs were found to be more environmentally friendly than their unmodified counterparts when they had the same or higher wood fiber (WF) content. SBS was viable to toughen the PLA-based WPCs from an environmental perspective. This cradle-to-gate LCA is likely to help optimize the manufacturing process and mitigate environmental impacts for the natural fiber-reinforced polymer biocomposites.

## 1. Introduction

Wood plastic composites (WPCs) made from petroleum-derived plastics, such as polyethylene and polypropylene, have been reported to have adverse impacts on the environment [1,2], since after their end of life they give rise to secondary pollutions [3]. To overcome the drawbacks of the traditional WPCs, bio-based and biodegradable polylactide (PLA) has been used as the matrix to manufacture renewable WPCs [4,5,6,7]. PLA-based biocomposites have excellent degradability, good processability, and tensile properties [8,9,10], since pristine PLA has distinguished degradability, processability, and environmental friendliness [11]. Beyond that, such PLA-based composites are perceived as more environmentally friendly than their fossil-derived counterparts.

However, the PLA-based WPCs suffer from inferior impact resistance due to the inherent brittleness of virgin PLA. Thus, some toughening agents were added to improve the impact strength of the resultant PLA-based WPCs. As far as mechanical properties are concerned, both polyhydroxyalkanoates (PHAs) [5] and styrene-butadiene-styrene (SBS) [6] have been verified to be excellent toughening agents for the PLA-based WPCs, according to our previous work. As opposed to the bio-based and bio-synthesized PHAs, however, SBS is one of the petroleum-derived agents. It maybe compromises the environmental friendliness of the resultant PLA-based WPCs when SBS is used as a toughening agent, even though it has a relatively low addition content. Consequently, the PLA-based WPCs modified with SBS should be assessed for their environmental friendliness before they are used as engineering materials for commercial applications.

Life cycle assessment (LCA) is an important tool to assess the environmental impacts of product systems and decisions. Some polymers, such as polystyrene (PS), polyethylene terephthalate (PET), and PLA have been investigated via this LCA method [12] beyond ethanol production [13], materials design [14], wastewater treatment, and industrial ecology [15,16]. Previous research concluded that global warming, non-renewable energy consumption, acidification, and eutrophication potentials of the natural fibers-reinforced polymer composites are superior to that of virgin polypropylene and polyethylene [17]. Also, the lignin-based automotive parts made through P4 technology offer the greatest life cycle energy and carbon dioxide emissions benefits [18], since the lignin comes from the by-products of ethanol and paper production.

Five life cycle stages are included in a whole LCA: acquisition of raw materials, product manufacture, packing and transport, product consumption/utilization, as well as the end-of-life waste disposal [19], which spans the cradle-to-grave life cycle. However, raw data of resource consumption, energy requirement, and the detailed pollutant emissions during the product usage and waste disposal stages are hard to collect for the LCA of composite materials [20]. Thus, a cradle-to-gate LCA is usually used to replace the cradle-to-grave LCA to assess its environmental impacts [21]. At this point, three life-cycle stages are included in the cradle-to-gate inventory: acquisition of raw materials, transportation, as well as product manufacture [22,23]. Previous research reported that global warming potential (GWP), energy use, and eutrophication potential (EP) of the bio-based paper binder (i.e., distiller grains) are comparable to those for the petroleum-based binder (i.e., polyvinyl alcohol) [22].

According to a previous work, the environmental impact load (EIL) of the PLA-based WPCs was reduced after being toughened with PHAs [24], which led to more environmentally friendly PLA-based WPCs. This means that toughening the PLA-based WPCs with PHAs is a win–win strategy, from the perspectives of both mechanical improvement and environmental benefits. The reason for its environmental benefits lies in how PHAs are a renewable, bio-based, bio-synthesized, and biodegradable polymer by nature [24]. For the PLA-based WPCs toughened with the fossil-derived SBS, its environmental impacts should also be comprehensively evaluated before it is used in commercial applications.

This work aims to investigate whether SBS is viable to be used to toughen the PLA-based WPCs, from an environmental perspective. Cradle-to-gate energy and resource inputs, as well as the pollutant emissions from one metric ton of the resultant PLA-based WPCs will be evaluated via the LCA inventory method. The following environmental impact categories: GWP, acidification potential (AP), EP, photochemistry oxidation potential (PCOP), smog potential (SP), and eco-toxicity potential (ETP) will be metered via the life cycle inventory method. Then, the environmental impacts of the PLA-based WPCs modified with SBS will be estimated according to their EIL index.

## 2. Materials and Methods

WF, PLA, and SBS were used as the filler, matrix, and toughening agent for the resultant WPCs, respectively. Pine wood (Dahurian larch) from Da Hinggan Ling Prefecture, Heilongjiang, were cut down, chopped off the branches, and air-dried close to the timberland. Then, the dried pine lumber was ground into WF. PLA (Injection grade, 1323) and SBS (YH-805, 4452) were purchased from Shenzhen, Guangdong and Yueyang, Hunan, respectively.

There were four different compositions of the PLA-based WPCs (i.e., Sample 1, 2, 3, and 4), subjected to this cradle-to-gate LCA. Sample 1 is the unmodified PLA-based WPCs, which was used as the reference. The content of PLA and WF in Sample 1 accounted for 80 and 20 wt %, respectively. Sample 2, 3, and 4 are the PLA-based WPCs toughened with SBS, with 5 wt % of adding content. WF content in Sample 2, 3, and 4 holds 10, 20, and 30 wt %, respectively [6]. Thus, the content of PLA in Sample 2, 3, and 4 is 85, 75, and 65 wt %, respectively.

**Figure 1:**
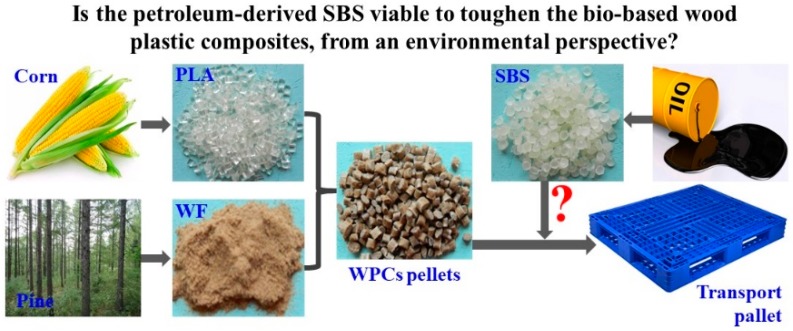
Raw materials and manufacture of the polylactide (PLA)-based wood plastic composites (WPCs) toughened with styrene-butadiene-styrene (SBS).

All the raw materials were transported to Xi’an, Shaan’xi to manufacture the PLA-based WPCs. The PLA-based WPCs pellets toughened with SBS were manufactured via extrusion blending. Then, these PLA-based WPCs pellets were used to produce transport pallets through injection molding [6] (Figure 1). The manufacture parameters were kept the same with our previous work.

**Figure 2:**
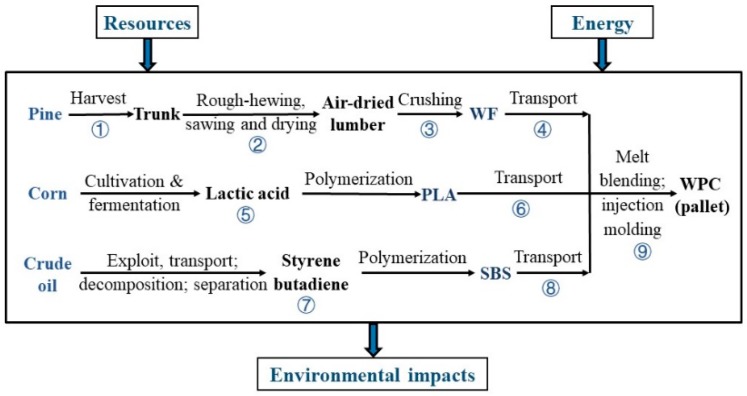
System boundary of this life cycle assessment (LCA) for the SBS-toughened, PLA-based WPCs.

The system boundary of this LCA research is schematically depicted in Figure 2. Energy consumption, water demand, as well as the pollution emissions from the binary PLA-based WPCs and their ternary SBS-modified counterparts will be investigated during their cradle-to-gate life cycles.

In Figure 2, Steps ① to ⑨ were used to describe the life cycle stages of the PLA-based WPCs modified with SBS. In this context, its cradle-to-gate inventory includes three main sections: acquisition of the raw materials (Steps ①–③, ⑤ and ⑦), transportation of the raw materials (Steps ④, ⑥, and ⑧), and product fabrication (Step ⑨). Thus, Steps ⑦ and ⑧ were excluded from Sample 1, since there was no SBS in the unmodified PLA-based WPCs.

During this LCA research, the PLA-based WPCs were used to manufacture transport pallets (or transport trays) for the logistic industry. Thus, the functional unit was set as one metric ton of transport pallets manufactured with the PLA-based WPCs. Neither the technological, economic, and social benefits, nor the quality, properties, cost, profit, and public image of the transport pallets manufactured with the PLA-based WPCs, were taken into consideration in this LCA research.

The life cycle inventory method was used to investigate energy and water demand, as well as the pollutant emissions from 1000 kg of the PLA-based WPCs [25]. Then, an input–output model was used to estimate environmental impacts for the PLA-based WPCs during its cradle-to-gate life cycles, as described in Bolin and Xu’s works [19,26].

As illustrated in Figure 2, acquisition of the raw materials was composed of Steps ①–③, ⑤, and ⑦ for this cradle-to-gate life cycle inventory. Access to the filler WF included pine harvest (Step ①), cutting off the branches and trunk drying (Step ②), and milling the pine lumber into WF (Step ③). Step ⑤ was made up of three parts: cultivation of maize, fermentation of the feedstock corn to access lactic acid, and polymerization of lactic acid into PLA. Step ⑦ covered the exploitation and extraction of crude oil, access to styrene and butadiene, and co-polymerization of them into SBS.

For Sample 1, the energy requirements, water consumption, and pollutant emissions during the raw materials acquisition stage was calculated by ((① + ② + ③) × 20% + ⑤ × 80%). For Samples 2, 3, and 4, they were formulated by (① + ② + ③) × 10% + ⑤ × 85%+ ⑦ × 5%, (① + ② + ③) × 20% + ⑤ × 75% + ⑦ × 5%, and (① + ② + ③) × 30% + ⑤ × 65% + ⑦ × 5%, respectively.

In this LCA research, the ratio of railway and road transportation to the total transport mileage was about 70% and 30%, respectively. The energy requirement for one kilometer of railway and road transportation was 0.0073 and 0.0844 kgce/t, respectively [27]. The energy requirement, water consumption, and pollution emissions during the transportation stage was calculated by: ④ × 20% + ⑥ × 80%, ④ × 10% + ⑥ × 85% + ⑧ × 5%, ④ × 20% + ⑥ × 75% + ⑧ × 5%, and ④ × 30% + ⑥ × 65% + ⑧ × 5% for Samples 1, 2, 3, and 4, respectively, according to their individual composition.

Different kinds of pollution have different effects on the environment. For example, CO_2_, CH_4_, and N_2_O contribute to GWP with different characterization factors, while SO_2_ contributes to AP, PCOP, and ETP. This study considers six different environmental impact categories from the PLA-based WPCs, including GWP, AP, EP, PCOP, SP, and ETP [28]. In this regard, each kind of environmental impact potential was measured with the weighted sum of different pollutant emissions and their individual characteristic factors [29].

However, different environmental impact potentials cannot be compared with each other directly for an LCA process, due to different physical units. In this case, all the six environmental impact potentials related to this LCA research were normalized to generate a non-dimensional index, EIL, to operate with each other directly [29]. Thus, researchers and decision-makers can use the EIL values to directly compare and evaluate their environmental performance of similar products.

Here, the analytic hierarchy process (AHP) method was used to determine the weighting factors of different pollutant emissions to their relevant environmental impact categories [30]. The advantage of this method is that it allows researchers and decision-makers to effectively combine experimental results with expert advice, and thus make a rational decision [31,32]. In this LCA research, pollution emissions of CO_2_, CO, SO_X_, CH_4_, NO_X_, N_2_O, NH_3_, volatile organic compounds (VOC), and particulate matter (PM) were set as the solution layer. The six environmental impact categories to be studied, i.e., GWP, AP, EP, SP, PCOP, and ETP, were set as the criterion layer. Thus, the EIL index was set as the target layer for the LCA of the PLA-based WPCs toughened with SBS [24].

An attribute hierarchy model (AHM) was used in order to determine the relative significance of different impact categories to the total EIL for the to-be-evaluated product in the local or regional scale, since different regions have different climate conditions [33]. It allowed for an evaluation on environmental impacts from different categories at one site [29], which facilitated the evaluation by enabling a wider and deeper understanding [15]. Here, the weighting coefficients of the above-mentioned environmental impact categories were kept the same as those in our previous research [24], since both the PHAs- and SBS-modified, PLA-based WPCs were manufactured in Xi’an, Shaanxi. Thus, the weighting coefficients of GWP, AP, EP, SP, PCOP, and ETP was 0.38, 0.25, 0.16, 0.064, 0.10, and 0.043, respectively, when they were used to calculate the total EIL for the PLA-based WPCs toughened with SBS.

## 3. Results and Discussion

### 3.1. Pollution Emissions

**Figure 3:**
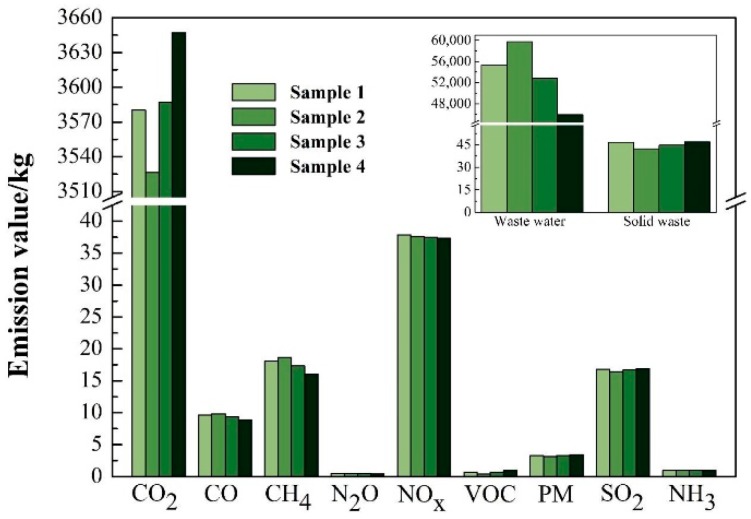
Waste emitted from one ton of the PLA-based WPCs.

The main pollutants emitted from one ton of the PLA-based WPCs during each step are summarized in an inventory shown in Table 1. All the raw data were excerpted from the previously published peer-reviewed literature. In this cradle-to-gate LCA, some pollutant emissions, from the processes with minimal outputs or hard-to-collect data, were omitted from the dataset and the following impact assessment. The weighting method was used to calculate the total emission of the individual pollutant, according to the different compositions of Sample 1 to 4. The results are shown in Figure 3.

#### 3.1.1. Waste Gases

CO_2_ keeps the largest emission among all the gas pollutants, since plenty of CO_2_ exhausts from one metric ton of the PLA-based WPCs toughened with SBS during the pallet manufacture phase (i.e., step ⑨), thus giving that the relative quantity—one ton of the PLA-based WPCs—produces more than 3.5 times the amount of CO_2_. For example, Sample 2 emitted 3527 kg of CO_2_ during its cradle-to-gate life cycle phase.

Also, CO_2_ emission had the greatest variation from Sample 1 to 4 among all the gas pollution. N_2_O and VOC held the least two among all the gas pollutions, yet with different trends. According to Figure 3, NOX, CH_4_, and SO_2_ constituted the middle level of waste gas emissions. This implies that there probably was substantial PCOP for the production process of these PLA-based WPCs. The classification and characterization of the waste gases, as well as the environmental impacts that they brought about, will be discussed in detail in the following sections.

#### 3.1.2. Water Consumption 

As shown in Figure 3, water demand for one ton of Sample 1 was 55 m^3^ during its cradle-to-gate profile. Their counterpart value for Sample 2, 3, and 4 was 60, 53 and 46 m^3^, respectively. Thus, both the unmodified and modified PLA-based WPCs required more than 45 m^3^ of water during their cradle-to-gate profiles. The reason for this lies in the fact that 69 m^3^ of water resource needs to be used to produce one ton of NatureWorks^®^ PLA, according to Vink’s finding [25]. This means that production of the PLA-based WPCs can save water resource, compared with pristine PLA.

When the PLA-based WPCs had the same content of WF (i.e., 20 wt %), one ton of the SBS-toughened WPCs (Sample 3) had a 4.4% decrease in water consumption, compared with their unmodified one (Sample 1). The reason for this is that Sample 3 had lower PLA content than Sample 1 (75% vs. 80%). Water consumption for Samples 2, 3, and 4 successively decreased, indicating that water consumption can be reduced by increasing WF content when the PLA-based WPCs are toughened with the same content of SBS. Beyond that, water consumption can be further reduced if the wastewater produced during the cradle-to-gate stage is recycled and reused after purification treatment. In this case, manufacturers of the PLA-based WPCs can reduce water consumption through one of the above-mentioned techniques in areas short of water resource.

#### 3.1.3. Solid Wastes

As shown in Figure 3, solid waste from one ton of Sample 1 was 46.7 kg during its cradle-to-gate scenarios. The counterpart value for Samples 2, 3, and 4 were 42.3, 44.6, and 46.9 kg, respectively. Acquiring WF produced the most amount of solid waste, followed by PLA acquisition. That is the reason why Sample 4 generated the most amount of solid waste among all four samples. During the WF acquisition stage, tree lumbering, piling, debarking, delimbing, and WF milling generated plenty of solid waste. In practice, a part or full amount of solid waste, such as residues in woodland and wood shavings from wood processing, was composted under natural conditions or incinerated to access energy. In this case, the solid waste emissions from or energy needed for the PLA-based WPCs were overstated in this cradle-to-gate LCA research.

### 3.2. Environmental Impacts

The environmental impact potentials of different pollutants to its corresponding environmental impact categories were metered by multiplying the pollutant emissions and their individual characteristic factors. The results are tabulated in Table 2.

The total amount of each kind of environmental impact was calculated by directly figuring up the environmental impact potentials of different pollution emissions together, as shown in Table 3. As far as the environmental impact potentials are concerned, GWP ranked the first among all the six environmental impact categories for all the PLA-based WPCs. However, GWP is a global environmental effect, while some environmental effects, such as PCOP, usually give rise to local or regional environmental threats. Thus, the non-dimensional index EIL was constructed to evaluate the environmental impacts of the given products, such as the PLA-based WPCs.

Global environmental impact potentials per capita in 1990 (POP90) was used as the benchmark to ensure the temporal identity for LCA [29]. This meant that the LCA findings in different regions of the globe could be compared with each other directly. Thus, the normalized environmental impact potentials were calculated by dividing the above summations of environmental impact potentials with the benchmark values. The normalized values are also presented in Table 3. 

**Figure 4:**
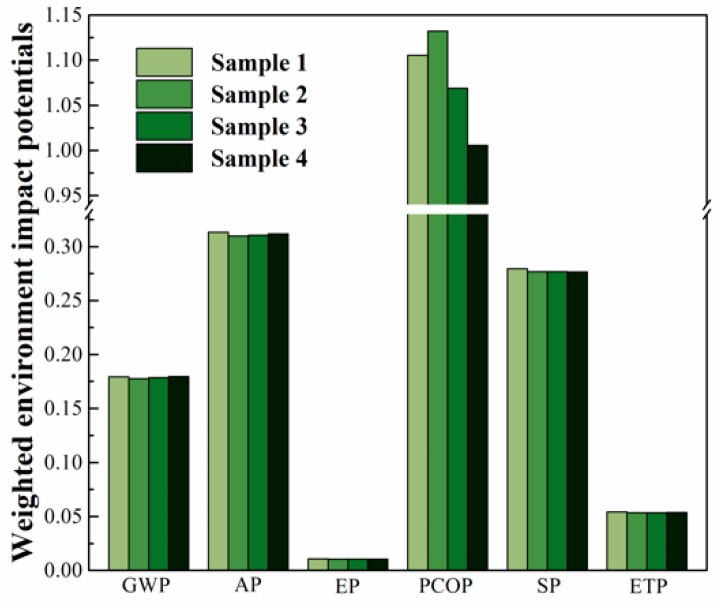
Weighted environmental impact potentials for the PLA-based WPCs during the cradle-to-gate stages.

The individual EIL of GWP, AP, EP, PCOP, SP, and ETP from one ton of Sample 1 to 4 was calculated by multiplying the normalized environmental impact potentials with their corresponding weighting coefficients. The results are shown in Figure 4. The PCOP took the largest share among all the six environmental impact categories for all the PLA-based WPCs (Sample 1 to 4), though the total environmental impact potential just added up to about 10, according to Supplementary file Table 3. However, the normalized environmental impact potentials were more than 15.5 for all the PLA-based WPCs, since the PCOP had the smallest POP90 benchmark value (i.e., 0.65 kg C_2_H_4_ eq.) in this LCA research. That made the PCOP value of Samples 1, 2, 3, and 4 reach up to 1.1055, 1.1322, 1.0688, and 1.0055, respectively. They are higher than the global average (i.e., 1.0). Meanwhile, the PCOP value for Samples 1 to 4 had the greatest variation among all six environmental impact categories. What’s more, these PCOP values strongly depend on the composition of the PLA-based WPCs. In this context, it is crucial for the manufacturers to optimize the compositions of these PLA-based WPCs in order to reduce their PCOP value due to local environmental impacts, even if the mechanical properties of these composite materials can meet with the application scenarios.

As far as the total EIL are concerned, the value of one ton of Samples 1, 2, 3, and 4 added up to 1.942, 1.960, 1.899, and 1.838, respectively. As shown in Figure 4, the percentage of POCP reached up to 56.9, 57.8, 56.3, and 54.7 for Samples 1, 2, 3, and 4, respectively. This means that the PCOP effect is the most important and most sensitive environmental concern when the PLA-based WPCs have been manufactured in Xi’an, Shaanxi. On the contrary, all the other EILs are lower than 0.33. EP had the least proportion among all the six impact categories, since the PLA-based WPCs release a small amount of NH3 and phosphate-containing wastes. The weighted environmental impact of GWP for all the PLA-based WPCs is less than 0.18. This means that, the environmental impacts of GWP, AP, EP, SP, and ETP from the PLA-based WPCs do not come to one third of the global average.

Samples 3 and 4 had a lower value of total EIL than Sample 1. This confirms that, from an environmental friendliness perspective, SBS is able to toughen the PLA-based WPCs. The EIL value for one ton of Samples 2 to 4 is very close to that of Sample 1. In other words, the SBS-modified PLA-based WPCs have approximately the same level of environmental friendliness as the unmodified one. Also, the PLA-based WPCs toughened with SBS had the same order of magnitude in the EIL value with that of the PLA-based WPCs toughened with PHAs [24]. The results thus confirm that toughening the PLA-based WPCs with the petroleum-derived SBS does not comprise the environmental benefits of the resultant composite materials. Furthermore, the total EIL value of the PLA-based WPCs modified with SBS decreases with the increasing WF content. This means that manufacturers can optimize the composition of these PLA-based WPCs to pursue the perfect balance between their mechanical properties and environmental benefits. However, the EIL value of the SBS-toughened, PLA-based WPCs may have been underestimated, since some of the SBS pollution emission was unavailable in this LCA research.

### 3.3. Energy Consumption

Energy consumption data for one ton of the PLA-based WPCs were excerpted from the previously published documents or professional reports, as shown in Table 4. Thus, their energy consumption could be metered across its cradle-to-gate profile according to the input–output model.

**Figure 5:**
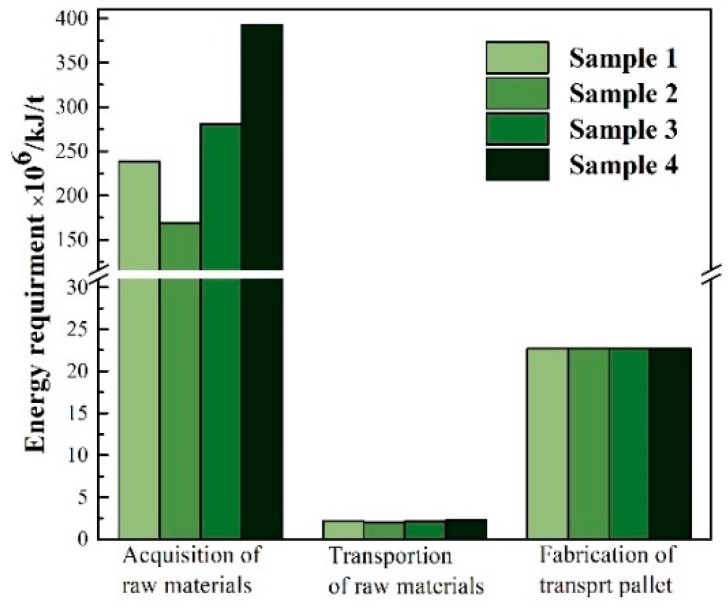
Energy demand for one ton of the PLA-based WPCs.

Energy consumption for one ton of the PLA-based WPCs was composed of three parts: acquisition of raw materials (WF, PLA, and SBS), transport of these raw materials, and manufacture of the transport pallets, according to the system boundary of this LCA research. Energy consumption for Samples 1 to 4 during their cradle-to-gate stages was metered, based on their individual compositions. The results are presented in Figure 5.

The total energy input for one ton of Samples 1, 2, 3, and 4 added up to 3.08 × 10^8^, 1.93 × 10^8^, 3.06 × 10^8^, and 4.18 × 10^8^ kJ, respectively. Among them, the energy used to acquire the raw materials for Samples 1, 2, 3, and 4 was 2.38 × 10^8^, 1.69 × 10^8^, 2.81 × 10^8^, and 3.93 × 10^8^ kJ, respectively. This means that the percentage of the total energy requirement accounted for 77.3, 87.6, 91.8, and 94.0, respectively. As far as the three raw materials are concerned, acquisition of WF required the majority of total energy, since the pine lumber air-drying consumed 99.6% of energy during the WF acquisition process. If the pine lumber were dried under natural conditions, Step ② could be excluded from the system boundary; thus, about 75% of energy could be saved. Energy used to fabricate transport pallets, that is, Step ⑨, was 22.7 × 10^6^ kJ/t [41], and energy used for transportation was 2.19 × 10^6^, 2.02 × 10^6^, 2.15 × 10^6^ and 2.28 × 10^6^ kJ for one ton of Samples 1, 2, 3, and 4, respectively. This accounted for only 0.71, 1.05, 0.70, and 0.55% of the total energy consumption, respectively.

Also, energy requirements for Samples 2, 3, and 4 increased with the increasing WF content, under the same SBS content. To save energy, acquisition of WF is the most crucial step. Energy consumption could greatly reduce if the processes for raw materials acquisition can be optimized in the future, such as if the lumber is dried under natural conditions, rather than under the current air-dried conditions.

## 4. Conclusions

In this LCA research, energy and water consumption, as well as the pollution emissions from the PLA-based WPCs and their SBS-toughened counterparts were investigated during the cradle-to-gate stages. The EIL value was calculated with a process-based input–output hybrid model and used as the index to evaluate environmental impacts of the resultant WPCs. The results showed that one metric ton of the SBS-toughened WPCs consumed at least 1.93 × 10^8^ kJ of energy (Sample 2) and 46 m^3^ of water (Sample 4), and produced 42.3 kg of solid waste (Sample 2). During the stage of acquiring raw materials, about 75% of energy was used to acquire WF, while most of the water was consumed to acquire PLA. In future, some viable measures should be taken, such as drying pine lumber under natural conditions and recycling wastewater and solid waste, to reduce the total energy and water consumption.

As far as the weighted environmental impact potentials are concerned, PCOP took the largest proportion among all the six environmental impact categories, while EP held the smallest proportion for one ton of the PLA-based WPC during the cradle-to-gate stages. Some measures should be taken to reduce their PCOP value, due to their local nature. The EIL index of Samples 1, 2, 3, and 4 was 1.942, 1.960, 1.899, and 1.838, respectively. Modifying the PLA-based WPCs with SBS was able to reduce their EIL index, compared with their untoughened counterparts. This indicates that the PLA-based WPCs toughened with SBS is more environmentally friendly than the untoughened ones when they have the same or higher WF content. However, the EIL value of the SBS-toughened PLA-based WPCs may have been underestimated, since the pollution emission data of SBS was not available in this research. The EIL of Samples 2, 3, and 4 reduced with the increasing WF content, which contributed to the favorable environmental benefits of WF.

This investigation provides useful information to policy makers, manufacturers, and consumers to further develop sustainable WPCs. Also, the findings can help to optimize eco-design and supply chain management to reduce the depletion of resources and pollutant emissions for PLA-based WPCs during their cradle-to-gate profiles. However, the LCA results can only be one of the references for them, because the weighting coefficients for different environmental impact categories mainly depend on expert advice. On the other hand, the cradle-to-gate evaluation of the PLA-based WPCs does not include product usage and end-of-life cycle stages, which maybe limits its ability to identify burden transfers. Some underestimation for environmental impacts, as well as energy and water consumption may exist in the LCA of the PLA-based WPCs, since pollutant emissions from some processes with minimal outputs were ignored from the inventory analysis. In future, the whole cradle-to-grave LCA and life-cycle cost (LCC) assessment will be comprehensively implemented for these composite materials modified with petroleum-based agents.

## Figures and Tables

**Table 1:** Cradle-to-gate inventory of the PLA-based WPCs.

Step	Waste Gases/kg									Waste Water/kg	Solid Waste/kg	Data Source
	CO_2_	CO	CH_4_	N_2_O	NO_X_	VOC	PM	SO_2_	NH_3_			
①+②+③	426.157	0.989	1.1007	0.0251	6.0998	3.072	1.368	4.72	0.00981	4.14	64.8	Ref [34,35], natural gas as fuel.
④	68.039	0.274	0.00262	0.00042	1.073	0.0664	0.04215	0.06039	n.a.	n.a.	n.a.	Ref [27,35]
⑤	−156.527	5.778	13.848	0.365	7.727	0.00024	0.0149	2.465	0.00492	69021^a^	42	Ref [25]
⑥	47.887	0.193	0.00184	0.0003	0.755	0.0467	0.02967	0.0425	n.a.	n.a.	n.a.	Same as ④
⑦	n.a.	n.a.	n.a.	n.a.	n.a.	n.a.	n.a.	n.a.	n.a.	20000^b^	n.a.	Ref [36]
⑧	24.932	0.1	0.00096	0.00015	0.393	0.0243	0.0154	0.0221	n.a.	n.a.	n.a.	Ref [37]
⑨	3568.394	4.639	6.746	0.187	29.652	n.a.	2.982	13.849	0.96	53.585	0.129	Ref [28,37]

Note: n.a. is the abbreviation for ‘not available’; a means there is 0.0119831 kg of phosphate included; b: 20,000 kg of process water required in this step.

**Table 2:** Environmental impact potentials of different pollutants from 1 ton of Sample 1 to 4.

Environmental Categories	Pollution	Characteriza-tion Factor ^c^	Environmental Impact Potentials			
			Sample 1	Sample 2	Sample 3	Sample 4
**GWP** kg CO_2 _eq./t	CO_2_	1	3580.321	3526.716	3587.000	3647.283
	CH_4_	21	378.987	391.209	364.434	337.659
	N_2_O	298	144.232	149.000	138.868	128.736
**AP** kg SO_2 _eq./t	SO_2_	1	16.811	16.460	16.687	16.914
	NO_x_	0.7	26.510	26.319	26.228	26.136
	Phosphate	0.98	0.012	0.012	0.012	0.012
	NH_3_	1.88	1.816	1.814	1.816	1.816
**EP** kg PO_4_^3-^eq./t	Phosphate	1	0.012	0.012	0.012	0.012
	NO_x_	0.1	3.787	3.760	3.747	3.734
	NH_3_	0.35	0.338	0.338	0.338	0.338
**PCOP** kg C_2_H_4 _eq/t.	CO	0.027	0.261	0.266	0.253	0.240
	NO_x_	0.03	1.136	1.128	1.124	1.120
	SO_2_	0.048	0.807	0.790	0.801	0.812
	CH_4_	0.5	9.024	9.315	8.677	8.040
**SP** kg PM eq./t	PM	1.014	3.346	3.206	3.344	3.483
	NO_x_	1.24	46.961	46.623	46.460	46.298
**ETP** kg 1,4-DCB eq./t	VOC	1	0.665	0.355	0.664	0.973
	SO_2_	0.096	1.614	1.580	1.602	1.624
	NH_3_	0.1	0.097	0.097	0.097	0.097
	NO_x_	1.2	45.446	45.119	44.962	44.804

Note: DCB: dichlorobenzene. eq. is the abbreviation for equivalents. c: characterization factor is quoted from Ref [29,38,39,40].

**Table 3:** Total and normalized environmental impact potentials.

Environmental Categories	Environmental Impact Potentials				Global Benchmark of Environmental Impact Potential per Capita in 1990^d^	Normalized Environmental Impact Potentials			
	Sample 1	Sample 2	Sample 3	Sample 4		Sample 1	Sample 2	Sample 3	Sample 4
GWP	4103.5	4066.9	4090.3	4113.7	8700 kg CO_2_ eq.	0.472	0.467	0.470	0.473
AP	45.1	44.6	44.7	44.9	36 kg SO_2_ eq.	1.253	1.239	1.242	1.247
EP	4.14	4.11	4.10	4.08	62 kg PO_4_^3−^eq.	0.0668	0.0663	0.0661	0.0658
PCOP	11.228	11.499	10.855	10.212	0.65 kg C_2_H_4_ eq.	17.274	17.691	16.700	15.711
SP	50.31	49.83	49.80	49.78	18 kg PM eq.	2.795	2.768	2.767	2.766
ETP	47.8	47.2	47.3	47.5	38 kg 1,4-DCB eq.	1.258	1.242	1.245	1.250

Note: d: Global benchmark of environmental impact potential per capita in 1990, cited from Ref [29].

**Table 4:** Energy requirements for 1 ton of the PLA-based WPCs.

Step	Energy Requirement ×10^6^/kJ/t	Dataset and Assumptions
①	2.09	Harvesting process includes 5 steps: felling trees, skidding trees to landing area, processing trees to logs (debarking, topping, bucking, delimbing and cutting to length), loading and transportation to the process point (hauling), Ref [35].
②	1174.32	3.6×50×551×0.404×29307.6=1174.32×10^6^ kJ/t. The moisture content of green heartwood reduces from 70% to 20%. The density of pine wood is 551 kg/m^3^, when its moisture is 20 %. 3.6 kWh/m^3^ is required for 1 % moisture reduction within the wood, Ref [39,40].
③	2.97	Natural gas as fuel, Ref [35].
④	3.22	(7.3×0.7+84.4×0.3)×3609×10^-3^×29307.6=3.22×10^6^ kJ/t. One kWh equals to 0.404 kgce, vice versa, 1 kgce equals to 29307.6 kJ. The distance of Great Khingan to Xi’an is 3,609 km (Great Khingan to Harbin is 989 km and Harbin to Xi’an is 2,620 km), Ref [27].
⑤	58.41	Nature Works PLA6, Ref [25].
⑥	1.93	(7.3×0.7+84.4×0.3)×2160×10^-3^×29307.6=1.93×10^6^ kJ/t. The distance from Shenzhen to Xi’an is 2,160 km.
⑦	20.9	Five hundred kWh of electricity and 5.6 t of steam are used for 1 ton of dry latex, equivalent to 20.9×10^6^ kJ/t, Ref [36].
⑧	1.18	(7.3×0.7+84.4×0.3)×1322×10^-3^×29307.6=1.18×10^6^ kJ/t. The distance from Yueyang to Xi’an is 1,322 km.
⑨	22.73	Energy requirement for extrusion blending and injection molding is 0.44 kWh/kg and 1.48 kWh/kg, respectively, Ref [41].
